# NKT Cells Contribute to the Control of Microbial Infections

**DOI:** 10.3389/fcimb.2021.718350

**Published:** 2021-09-14

**Authors:** Stefan Vogt, Jochen Mattner

**Affiliations:** ^1^Mikrobiologisches Institut - Klinische Mikrobiologie, Immunologie und Hygiene, Universitätsklinikum Erlangen and Friedrich-Alexander Universität (FAU) Erlangen-Nürnberg, Erlangen, Germany; ^2^Medical Immunology Campus Erlangen, FAU Erlangen-Nürnberg, Erlangen, Germany

**Keywords:** NKT cells, lipid antigens, cognate activation, tissue homeostasis, microbial infection, bystander activation

## Abstract

Innate (-like) T lymphocytes such as natural killer T (NKT) cells play a pivotal role in the recognition of microbial infections and their subsequent elimination. They frequently localize to potential sites of pathogen entry at which they survey extracellular and intracellular tissue spaces for microbial antigens. Engagement of their T cell receptors (TCRs) induces an explosive release of different cytokines and chemokines, which often pre-exist as constitutively expressed gene transcripts in NKT cells and underlie their poised effector state. Thus, NKT cells regulate immune cell migration and activation and subsequently, bridge innate and adaptive immune responses. In contrast to conventional T cells, which react to peptide antigens, NKT cells recognize lipids presented by the MHC class I like CD1d molecule on antigen presenting cells (APCs). Furthermore, each NKT cell TCR can recognize various antigen specificities, whereas a conventional T lymphocyte TCR reacts mostly only to one single antigen. These lipid antigens are either intermediates of the intracellular APC`s-own metabolism or originate from the cell wall of different bacteria, fungi or protozoan parasites. The best-characterized subset, the type 1 NKT cell subset expresses a semi-invariant TCR. In contrast, the TCR repertoire of type 2 NKT cells is diverse. Furthermore, NKT cells express a panoply of inhibitory and activating NK cell receptors (NKRs) that contribute to their primarily TCR-mediated rapid, innate like immune activation and even allow an adaption of their immune response in an adoptive like manner. Dueto their primary localization at host-environment interfaces, NKT cells are one of the first immune cells that interact with signals from different microbial pathogens. Vice versa, the mutual exchange with local commensal microbiota shapes also the biology of NKT cells, predominantly in the gastrointestinal tract. Following infection, two main signals drive the activation of NKT cells: first, cognate activation upon TCR ligation by microbial or endogenous lipid antigens; and second, bystander activation due to cytokines. Here we will discuss the role of NKT cells in the control of different microbial infections comparing pathogens expressing lipid ligands in their cell walls to infectious agents inducing endogenous lipid antigen presentation by APCs.

## 1 Introduction

Innate (-like) or unconventional T lymphocytes consist of a highly diverse group of cells. These include subsets of γ/δ T cells, mucosal-associated invariant T (MAIT) cells and natural killer T (NKT) cells (Godfrey et al., 2015; Legoux et al., 2017). During maturation in the thymus, they acquire characteristics specific for memory cells and exhibit unique trafficking patterns into peripheral, frequently non-lymphoid barrier tissues including the gut, the skin, the liver or the lung (Godfrey et al., 2015). There, innate (-like) T lymphocytes become resident, regulate tissue homeostasis or fight infections (Legoux et al., 2017). In contrast to conventional T lymphocytes, these innate (-like) T cell subsets express TCRs that are not restricted to the MHC-mediated presentation of peptide antigens (Legoux et al., 2017). Instead, MAIT cells, for example, recognize bacterial metabolite intermediates (Legoux et al., 2020) whereas NKT cells react to a variety of different self- or microbial lipid antigens, presented by the MHC-like molecules, MR1 or CD1, respectively (Godfrey et al., 2004; Bendelac et al., 2007; Singh et al., 2018). Following cognate TCR engagement, all three unconventional T cell subsets rapidly release copious amounts of cytokines and chemokines (Godfrey et al., 2004; Bendelac et al., 2007). Indeed, one single unconventional T cell clone can respond to various ligands, in contrast to the single antigen specificity of conventional T cells. Thus, the TCRs of innate (-like) T lymphocytes act rather as a pattern recognition receptor of the innate arm of the immune system than as highly diverse antigen recognition receptors of the adaptive arm of the immune system. Being frequently localized at interfaces of the host with its environment, innate (-like) T cells sense infections, form the first line of defense against invading pathogens and boost the subsequent innate and adaptive immune response.

In this review, we will focus on NKT cells (Godfrey et al., 2004; Bendelac et al., 2007; Godfrey et al., 2015; Singh et al., 2018).

## 2 NKT Cells

Natural killer T (NKT) cells belong to a subset of innate-like T lymphocytes that share properties of both, T and natural killer (NK) cells (Lantz and Bendelac, 1994; Bendelac et al., 1995; Godfrey et al., 2004). There are two main NKT cell subsets, which both react to a broad variety of endogenous and microbial, lipid-based antigens presented by the atypical MHC-I (-like) molecule CD1d on antigen presenting cells (APCs) (Bendelac et al., 2007; Birkholz and Kronenberg, 2015). Indeed, the best-characterized NKT cell subset are type 1 NKT cells, which express a semi-invariant TCR that combines Vα14-Jα18 with Vβ2, 7, 8 in mice and Vα24-Jα18 with Vβ11 in humans. Furthermore, all type 1 NKT cells react to the glycosphingolipid antigen alpha-galactosylceramide (α-GalCer) (Bendelac et al., 2007; Godfrey et al., 2018). In contrast, type 2 NKT cells exhibit a diverse TCR repertoire, do not react to α-GalCer and are more abundantly present in humans than in mice (Exley et al., 2001; Godfrey et al., 2018; Singh et al., 2018). Like other types of innate-like T lymphocytes, NKT cells are reactive to both, foreign and self-antigens (Bendelac et al., 2001). Following TCR engagement NKT cells can rapidly secrete high amounts of several cytokines and chemokines (Bendelac et al., 2007; Coquet et al., 2008). Second, NKT cells also express a wide range of activating and inhibitory NK cell receptors (Bendelac et al., 2007). While the role of activating NK cell receptors on NKT cell biology is only partially understood the inhibitory NK cell receptors control the self-reactivity of NKT cells and thus, avoid autoimmune activation (Bendelac et al., 2001; Kronenberg and Rudensky, 2005). Conversely, the NKT cell TCR also shapes the distribution of NK cell receptors (Skold et al., 2003; Voyle et al., 2003).

Following selection, maturation, cytokine polarization and egress from the thymus [for further reading please refer to (Kronenberg and Gapin, 2002; Bendelac et al., 2007; Constantinides and Bendelac, 2013)], NKT cells home to several lymphoid and non-lymphoid organs, including the adipose tissue, intestines, liver, lungs, lymph nodes and the spleen (Crosby and Kronenberg, 2018). NKT cells can exit the thymus either already as pre-committed subsets or can also acquire polarized functions in the periphery (Cameron and Godfrey, 2018). Once arrived at these distinct tissue sites, NKT cells usually reside there as non-circulating long-term residents and release a Th1, Th2, Th10 or Th17 polarized cytokine pattern following activation (Constantinides and Bendelac, 2013; Lee et al., 2013; Sag et al., 2014; Cameron and Godfrey, 2018). Although the substantial functional heterogeneity of polarized NKT cell subsets is well studied (Constantinides and Bendelac, 2013; Cameron and Godfrey, 2018; Crosby and Kronenberg, 2018), the factors and mechanisms underlying their recruitment to different tissues have not been characterized yet.

In humans, there exist large inter-individual variations in NKT cell numbers, ranging usually from 0.01% up to 1%, and in rare cases for up to 5% of the total T cell population in human blood, for example (Mattner, 2013; Godfrey et al., 2015). NKT cells comprise about 0.5% of the local T cell population in the blood and peripheral lymph nodes, around 2% in the spleen and up to 30% in the livers in mice (Benlagha et al., 2000; Matsuda et al., 2000; Hammond et al., 2001; Bendelac et al., 2007). Particularly, NKT cells accumulate in the livers of mice; there they patrol liver sinusoids for microbial and nutritional antigens (Geissmann et al., 2005) floating in from the portal vein system that drains the gastrointestinal tract. Although NKT cells contribute to the control of microbial infections, their aberrant activation under certain circumstances can also perpetuate tissue damage (Mattner, 2013).

## 3 NKT Cell Antigens

In contrast to conventional T cells, which recognize peptides bound to major histocompatibility complex (MHC) proteins, NKT cells react to lipid antigens presented by the MHC-like protein CD1d (Beckman et al., 1994; Brigl and Brenner, 2004). CD1d is a member of the CD1 family, which comprises five CD1 genes, CD1a, CD1b, CD1c, CD1d and CD1e in humans (Bradbury et al., 1988; Godfrey et al., 2018) with mice containing two orthologues of the CD1d gene, CD1d1 and CD1d2 (Bradbury et al., 1988; Balk et al., 1991). Both encode CD1d proteins, which are assembled in the endoplasmic reticulum, loaded with different microbial or self-antigens in the late endosome and delivered to the cell surface (Bricard and Porcelli, 2007; Cohen et al., 2009). There CD1d presents these antigens to NKT cells. Isoglobotrihexosylceramide (iGb3) (Zhou et al., 2004; Zajonc et al., 2008) and α-GalCer (Kain et al., 2014) have been suggested as endogenous ligands that are pivotal for the selection and differentiation of NKT cells in the thymus and their function in the periphery (Sundararaj et al., 2018). Furthermore, the CD1-restricted TCRs of NKT cells are autoreactive to CD1 expressing APCs (Cardell et al., 1995; Skold et al., 2003; Almeida et al., 2019). Indeed, endoplasmic reticulum (ER) stress in APCs is a potent inducer of CD1d-dependent NKT cell auto-reactivity (Govindarajan et al., 2020).

Synthetic α-GalCer was the first known antigen presented by CD1d that could stimulate the invariant TCR expressed by NKT cells (Kawano et al., 1997). This prototypical NKT cell ligand is a very close structural analog of several agelasphins, which have been isolated from extracts of the *Agelus* genus of marine sponges in the Okinawan sea because of their anti-tumor properties (Kobayashi et al., 1995; Morita et al., 1995). Importantly, α-GalCer is even active in picomolar concentration ranges. The physiological relevance of α-GalCer, however, was not well understood for many years. Nowadays, however, it is clear that α-GalCer or related structures are not only detected in marine sponges, but also in commensal or environmental bacteria (Kinjo et al., 2005; Mattner et al., 2005; Wieland Brown et al., 2013). Similarly, related structures have been identified in mammalian tissues or cow´s milk including α-psychosines (Kain et al., 2014; Kain et al., 2015; Deng et al., 2017) or α-glucosylceramides (GlcCers) (Brennan et al., 2014; Brennan et al., 2017). Many research groups have identified other microbial and endogenous NKT cell antigens. These include glycolipids, glycosphingolipids, diacylglycerols, glycerophospholipids, lysophospholipids and cholesterol esters (Mattner, 2013).

### 3.1 Microbial Antigens

#### 3.1.1 Bacterial Antigens

One of the bacteria in whose cell wall antigens for NKT cells have been detected were *Sphingomonas* spp. (Kinjo et al., 2005; Mattner et al., 2005; Sriram et al., 2005). The genus *Sphingomonas* and the three closely related new genera, *Sphingobium*, *Novosphingobium* and *Sphingopyxis* contain more than 30 different species (Takeuchi et al., 2001). All four *Sphingomonas* genera are ubiquitous α-proteobacteria, commonly found in the environment including soil, sediments, plants and water (Barbeau et al., 1996; Cavicchioli et al., 1999; Brodie et al., 2007). Furthermore, *Sphingomonas* spp. can colonize mucosal surfaces of humans and mice (Selmi et al., 2003; Ivanov et al., 2009; Wei et al., 2010; Huang et al., 2011). Opportunistic nosocomial and even community-acquired infections, albeit rarely have been reported for these non-fermentative, aerobic, gram-negative bacilli (Crane et al., 1981; Freney et al., 1987; Reina et al., 1991; Hsueh et al., 1998; Marinella, 2002; Kampfer et al., 2005; Kilic et al., 2007; Ryan and Adley, 2010; Lin et al., 2010; Toh et al., 2011; Lanoix et al., 2012). Furthermore, *Sphingomonas* spp. have been associated with primary biliary cirrhosis (Selmi et al., 2003; Kaplan, 2004; Padgett et al., 2005; Mattner et al., 2008; Mohammed and Mattner, 2009; Mohammed et al., 2011), a chronic cholestatic liver disease characterized by a T cell mediated destruction of small bile ducts and autoantibodies targeting the E2 subunit of the mitochondrial pyruvate dehydrogenase complex (PDC-E2) (Gershwin et al., 2000; Kaplan and Gershwin, 2005).

Instead of expressing lipopolysaccharide (LPS) in their cell wall, these Gram-negative bacteria abundantly express glycosphingolipids (GSLs) which exhibit strong structural similarities with α-GalCer when present as monosaccharides (GSL-1´) (Kawahara et al., 1990; Kawahara et al., 1991; Kawahara et al., 1999; Kawahara et al., 2000; Kawahara et al., 2001; Kawahara et al., 2002; Kawahara et al., 2006). Thus, it is not surprising, that NKT cell-deficient mice clear infections with *Sphingomonas* spp. less efficiently compared to respective littermate controls (Kinjo et al., 2005; Mattner et al., 2005). Dependent on the sugar moiety linked to the ceramide portion, these GSL-1s contain a glucuronic acid or a galacturonic acid and thus, are called α- glucuronosylceramide (GSL-1) or α-galacturonosylceramide (GSL-1´). Besides of being linked to one sugar, GSLs of *Sphingomonas* can also contain additional saccharide groups. These include tri-and tetrasaccharides entitled as GSL-3 and GSL-4, respectively. While GSL-1 and GSL-1´ activate NKT cells *in vitro* and *in vivo* (Kinjo et al., 2005; Mattner et al., 2005; Sriram et al., 2005), GSL-3 and GSL-4 are not antigenic (Long et al., 2007; Kinjo et al., 2008). As bacterial symbionts frequently colonize the *Agelus* genus of marine sponges (Dieckmann et al., 2005), the prototypical ligand α-GalCer itself might have originated from *Sphingomonas* or related bacteria.

Other bacterial NKT cell antigens are diacylglycerols (DAGs) which are expressed in the cell wall of *Borrelia burgdorferi*, the causative agent of Lyme disease, or *Streptococcus pneumonia*, a Gram-positive bacterium triggering meningitis or pneumonia (Kinjo et al., 2006; Kinjo et al., 2011). Indeed, DAGs from both bacteria directly stimulated NKT cells in a CD1d-retricted manner. Consistently, the anti-microbial defense against both pathogens *in vivo* was impaired in the absence of NKT cells (Kumar et al., 2000; Nakamatsu et al., 2007; Christaki et al., 2015). Moreover, NKT cells modulate also the severity of Lyme disease, even after the infection has been cleared (Tupin et al., 2008; Olson et al., 2009). Other diacylglycerols (DAGs) are found in the cell walls of *Corynebacterium glutamicum*, an uniquitous soil bacterium without clinical relevance, *Mycobacterium tuberculosis*, *Listeria monocytogenes* and *Mycobacterium smegmatis*, a fast-growing, non-pathogenic bacterial species usually detected in urogenital secretions. Interestingly, α-glucuronosyl diacylglycerol (α-GlcA-DAG) derived from *Mycobacterium smegmatis* stimulated a NKT cell subset carrying a semi-invariant α-chain Vα10-Jα50^+^  (Uldrich et al., 2011; Burugupalli et al., 2020) as well as type 2 NKT cells (Almeida et al., 2019). Although Vα10-Jα50^+^ NKT cells recognized α-GalCer presented by CD1d, they neither fit in the type 1 nor the type 2 NKT cell category. Interestingly, Vα10-Jα50^+^ NKT cells produced 10- to 100-fold more IL-4, IL-13 and IL-17 compared to type 1 NKT cells following stimulation with α-GlcA–DAG (Uldrich et al., 2011). Furthermore, the TCRs of Vα10-Jα50^+^ and type 2 NKT cells can bind with diverse docking modes to CD1d-antigen complexes (Almeida et al., 2019).

Bacterial derived phospolipids also stimulate NKT cells with diverse TCRs (Wolucka et al., 1993; Cao et al., 2013). For example, phosphatidylglycerol (PG), diphosphatidylglycerol (DPG) and phosphatidylinositol from *Mycobacterium tuberculosis*, *Listeria monocytogenes* or *Corynebacterium glutamicum* stimulated type 2 NKT cells in a CD1d-restriced manner (Tatituri et al., 2013; Wolf et al., 2015). Type 2 NKT cells protected also against Methicillin-resistant *Staphylococcus aureus* infections due to the recognition of polar bacterial lipid species containing both PG and lyso-PG (Genardi et al., 2020).

Furthermore, phosphatidylinositolmannoside PIM4 in *Mycobacterium tuberculosis* (Fischer et al., 2004) and cholesteryl α-glucosides in *Helicobacter pylori* (Chang et al., 2011; Ito et al., 2013), a causative agent of gastritis and malignant tissue transformation, have been suggested as NKT cell antigens. However, only purified, but not synthetic PIM4 of *Mycobacterium tuberculosis* activated NKT cells (Fischer et al., 2004; Kinjo et al., 2006). In addition, NKT cells played no significant beneficial role in clearing both bacteria (Behar et al., 1999; Chang et al., 2011). Thus, the biological relevance of both antigens remains unclear. Compared to other antigens, the abundance of NKT cell ligands in the cell wall of both bacteria might be lower. Moreover, the stimulatory capacity of PIM4 and cholesteryl α-glucosides has not been compared yet with the exception of a study testing few NKT cell clones (Burugupalli et al., 2020) to the one of GSLs or DAGs found in other bacteria. Nonetheless, we speculate that the NKT cell TCR might have evolved as a pattern recognition receptor for distinct classes of bacteria carrying GSLs or DAGs in their cell walls.

Interestingly, bacteria, such as *Bacteroides* spp., physiologic commensals of mucosal surfaces can also evade the recognition by NKT cells. Following trauma or surgery, this predominant genus of the gastrointestinal tract can convert into pathobionts and can also cause life-threatening infections. First, *Bacteroides* spp., prominent members of the human gut microbiota, produce α-GalCer (Wieland Brown et al., 2013). Compared to marine-sponge derived α-GalCer, however, this bacterial α-GalCer triggered significantly less IFN-γ release, suggesting that molecular differences in the respective ligand determine the level and type of NKT cell activation. Furthermore, *Bacteroides* spp. contain unusual membrane glycosphingolipids (GSLs) (Kato et al., 1995), which among other functions (An et al., 2011) impede the activation of NKT cells *in vitro* and *in vivo*, in particular, GSL-Bf717 of *Bacteroides >fragilis* (An et al., 2014). Thus, the blockade of NKT cell activation likely hampers the detection of this opportunistic pathogen. Furthermore, this unique ligand also shapes the homeostasis of intestinal NKT cells during neonatal development (An et al., 2014) suggesting that intestinal microbiota significantly impact NKT cell biology.

Indeed, there is evidence that intestinal microbiota and NKT cells mutually interact with each other (Nieuwenhuis et al., 2009; Wei et al., 2010; Olszak et al., 2012; Wingender et al., 2012). Relative and absolute numbers of NKT cells specifically increase in intestinal tissues of germ free (GF) mice (Olszak et al., 2012; Wingender et al., 2012). The accumulation of NKT cells in the absence of intestinal microbiota, particularly in the colonic mucosa, is due to an increased expression of CXCL16, the cell surface ligand for the chemokine receptor CXCR6 (Olszak et al., 2012) expressed on NKT cells (Johnston et al., 2003; Geissmann et al., 2005). Moreover, NKT cells from GF mice were hyperreactive following induction of experimental colitis and perpetuated intestinal pathology (Olszak et al., 2012). Subsequently, mutual interactions between microbiota and NKT cells maintain intestinal immune homeostasis and prevent mucosal damage (Zeissig and Blumberg, 2013). Interestingly, albeit CXCR6^-/-^ mice exhibit selectively reduced NKT cell numbers in the liver (Geissmann et al., 2005) and microbial antigens from the gut also circulate through the portal vein system into hepatic tissues, the absence of the intestinal microbiota did not significantly alter the expression of CXCL16 in hepatic tissues and thus, the accumulation of NKT cells there.

#### 3.1.2 Fungal Antigens

In addition to bacterial cell wall antigens, NKT cells also directly detect fungal GSLs. In contrast to the α-linked bacterial ligands, however, the prototype of a fungal GSL is a β-linked glucosylceramide, asperamide B. Asperamide B has been identified in *Aspergillus* (Albacker et al., 2013), a saprophytic mold that causes invasive aspergillosis in immunocompromised patients (Dagenais and Keller, 2009) and allergic sensitization in predisposed, but otherwise healthy individuals (Knutsen et al., 2012). Indeed, purified and synthetic asperamide B directly activate mouse and human NKT cells in a CD1d-restricted manner. Furthermore, mice experienced airway hyperreactivity, a characteristic feature of asthma, following exposure to asperamide B *in vivo* (Albacker et al., 2013). Thus, as most protein allergens can bind lipids (Thomas et al., 2005; Karp, 2010), NKT cells activated by the GSLs of *Aspergillus* likely enhance an allergic sensitization to accompanying proteins and the subsequent adaptive Th2 responses. In addition, alpha-glycosyl diacylglyceride of *Aspergillus fumigatus* (Fontaine et al., 2009) are also recognized by type 1 and type 2 NKT cells (Burugupalli et al., 2020).

Next to the cognate recognition of fungal cell wall antigens, bystander mechanisms trigger also NKT cell activation during fungal infections. For example, major fungal cell-wall polysaccharides such as β-1,3 glucans trigger IL-12 production in a dectin-1 and MyD88-dependent manner by APCs. This drives the self-reactive iNKT activation and the release of IFN-γ in response to *Aspergillus*, *Candida*, *Histoplasma* and *Alternaria* (Cohen et al., 2011). Furthermore, CD1d-deficient mice poorly control infections with *Aspergillus fumigatus* (Cohen et al., 2011) reflecting the need for NKT cells in the control of fungal infections. In addition, human NKT cells exhibit immunomodulatory effects following exposure to *Aspergillus* spp (Beitzen-Heineke et al., 2016).

#### 3.1.3 Protozoan Parasite Antigens

The third kingdom of microorganisms from which NKT cell antigens have been isolated from were protozoan parasites. For example, inositol and its derivatives have been proposed as NKT cell antigens. For example, the glycosylphosphatidylinositol (GPI) anchors of the surface proteins of *Plasmodium falciparum* or *Trypanosoma brucei*, the causative agents of malaria or African sleeping sickness, respectively, expanded NKT cells in a CD1d dependent manner (Schofield et al., 1999). These data, however, are controversial, as a subsequent study failed to induce NKT cell activation by GPI anchors (Molano et al., 2000). Furthermore, phosphoinositol (PI) antigens of *Entamoeba histolytica*, the causative agent of amoebiasis, also activated NKT cells (Lotter et al., 2009). In addition, the surface glycoconjugate lipophosphoglycan as well as related glycoinositol phospholipids of *Leishmania*, the protozoan parasite causing cutaneous und mucosal leishmaniasis, bind with high affinity to CD1d and induce a CD1d-dependent release of IFN-γ in subsets of intrahepatic lymphocytes (Amprey et al., 2004).

### 3.2 Endogenous Antigens

NKT cells are autoreactive towards CD1d-expressing cells (Brigl and Brenner, 2004). Endogenous ligands presented by CD1d presumably underlie this low-level auto-reactivity of NKT cells (Cardell et al., 1995; Burdin et al., 1998; Behar et al., 1999; Brossay and Kronenberg, 1999; Zhou et al., 2004; Chang et al., 2008). Since these ligands are potentially weaker agonists than microbial antigens, NKT cells require additional signals from other receptors and/or cell subsets for optimal activation when exposed to endogenous, CD1d presented ligands. Alternatively, the infection of APCs or different pathologic conditions increase or decrease the expression of CD1d molecules on their surfaces and subsequently, the amount and frequency of self-antigens presented to NKT cells resulting in altered NKT cell activation (Durante-Mangoni et al., 2004; Falcone et al., 2004; Yuan et al., 2006). Importantly, in contrast to the α-linked antigens from bacteria and protozoan parasites, a β-linkage of the sugar head to the ceramide lipid portion naturally occurs in mammals. Although several antigens have been described as endogenous ligands (Birkholz and Kronenberg, 2015), particularly isoglobotrihexosylceramide (iGb3) (Zhou et al., 2004; Zajonc et al., 2008) and β-glucosylceramide (Stanic et al., 2003; Brennan et al., 2011) that might be involved in NKT cell activation during bacterial infection (Mattner et al., 2005; Brennan et al., 2011). There is also evidence that infections lead to an accumulation self-lipid reactive type 1 and type 2 NKT cells (Mattner et al., 2005; Chang et al., 2008; Nair et al., 2015). However, whether one or both of these endogenous antigens or other ligands drive autoreactive NKT cell responses in infections with viruses, fungi or protozoan parasites is still largely unknown. Thus, the exact context in which endogenous ligand(s) trigger NKT cell activation still require definition. Furthermore, dependent on the tissue and the (patho-) physiological context, different self-antigens might be presented to NKT cells that likely affect their biology and subsequent functional response.

## 4 Mechanisms of NKT Cell Activation During Infection

**Figure 1:**
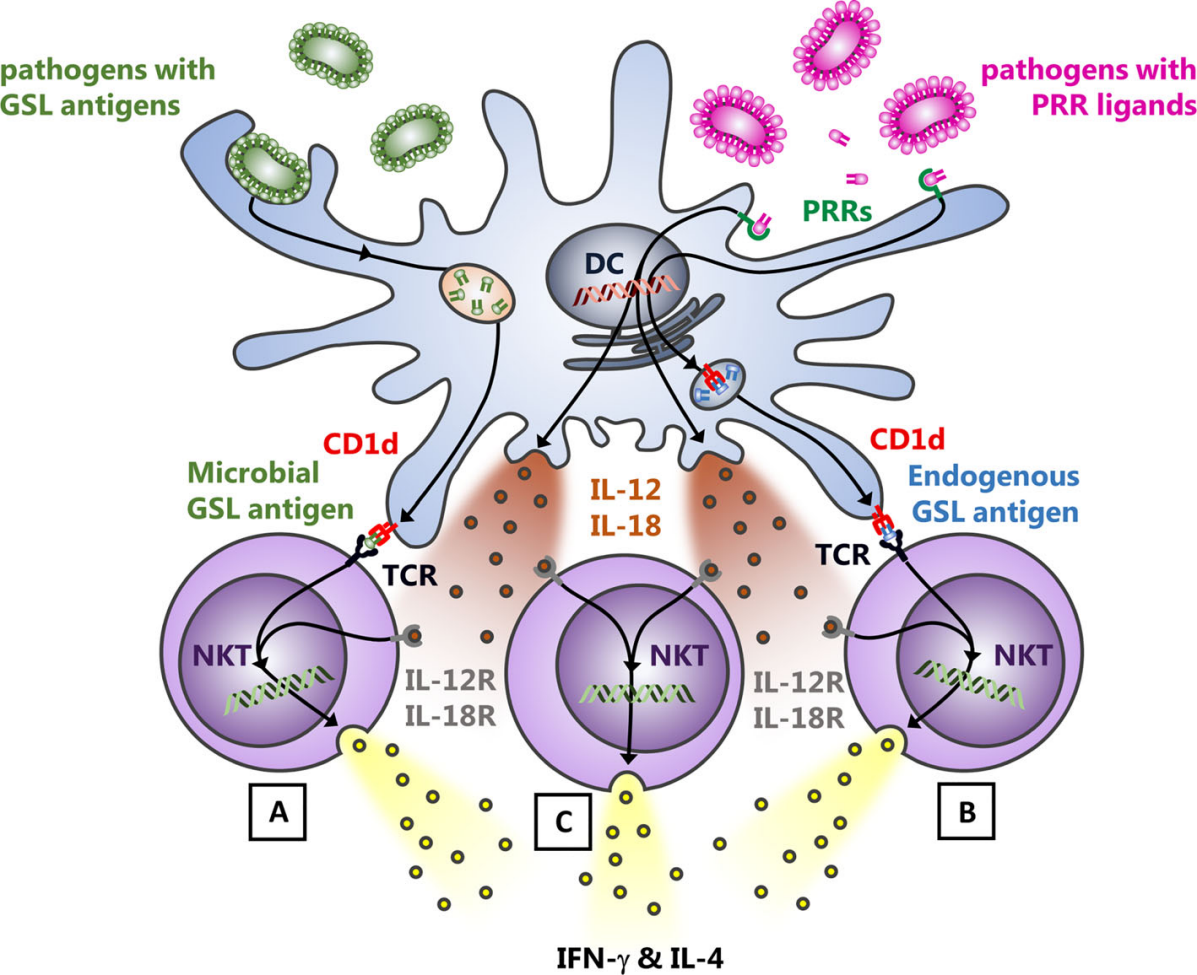
Modes of NKT cell activation upon bacterial infection. Following the uptake and digestion of bacteria by myeloid cells, three mechanisms predominantly drive the activation of NKT cells: **(A)**, bacterial cell wall glycosphingolipid (GSL) antigens presented by APCs are sufficient to induce the release of IFN-γ and IL-4 by NKT cells due to the CD1d mediated presentation of the ligand to the NKT cell TCR. **(B)**, endogenous GSL presentation *via* CD1d to the NKT cell TCR in response to infection and subsequent augmented NKT cell auto-reactivity. The identity of the lipid ligands underlying the auto-reactivity of NKT cells in different tissues and under distinct (patho-) physiological circumstances, however, still requires definition. **(C)**, the cytokine driven, TCR-independent activation of NKT cells for which TLR- oder Dectin triggered IL-12 and IL-18 release by APCs is responsible. This also further enhances the release of cytokines by NKT cells, activated by cognate TCR engagement [mechanisms **(A, B)**]. Thus, NKT cells are activated during infection with bacteria that do not express themselves NKT cell antigens in their cell walls or unable to induce endogenous antigens.

Following phagocytosis and degradation of microbial pathogens by different APCs and phagocytes, microbial and endogenous antigens and ligands are released which engage their respective receptors. Among those, lipid antigens are loaded onto CD1d molecules in the late endosome, which recycle afterwards back to the cell surface. There, NKT cells survey CD1d molecules for antigens as well as for additional accessory signals provided by APCs, which allow subsequent NKT cell activation. Based on the nature of these interactions, there are three major mechanisms driving NKT cell activation during microbial infection (**Figure 1**): A) NKT cells directly recognize due to cognate TCR engagement microbial lipid antigens presented by CD1d (Mattner et al., 2005; La Gruta et al., 2018); B) NKT cell TCRs react to endogenous ligands incorporated into CD1d; the availability of endogenous lipid antigens can be induced by pattern recognition receptor- (PRR-) driven APC activation (Mattner et al., 2005; Zeissig et al., 2012); C) engagement of toll-like receptors (TLRs) and Dectin by LPS, CpG or β-1,3-glucan drives the release of IL-12 and IL-18 by APCs, which leads to a TCR-independent activation of NKT cells (bystander activation) (Leite-De-Moraes et al., 1999; Brigl et al., 2011; Cohen et al., 2011; Moran et al., 2011; Holzapfel et al., 2014). This mechanism can also enhance the cognate TCR activation by exogenous or endogenous antigens (mechanisms A & B). However, IL-12, IL-18, and TLRs are completely dispensable for the TCR activation pathway of NKT cells when a strong TCR agonist is used (Anderson et al., 2021).

Accordingly, NKT cells activated by microbial glycolipid antigens contribute to bacterial clearance (cognate activation) (Kinjo et al., 2005; Mattner et al., 2005; Kinjo et al., 2006; Kinjo et al., 2011). However, the TLR-elicited cytokine responses of myeloid cells infected by bacteria that do not contain lipids in their cell wall also activate and recruit NKT cells as part of an inflammatory cellular network (bystander activation) (Brigl et al., 2003; Mattner et al., 2005). Furthermore, these myeloid cell-derived cytokines also enhance the TCR-mediated activation of NKT cells. Thus, an absence of NKT cells has not only been associated with an enhanced susceptibility to infectious diseases in mice (Kinjo et al., 2005; Mattner et al., 2005; Kinjo et al., 2006; Kinjo et al., 2008; Cohen et al., 2011), but also in humans. Indeed, genetic mutations resulting in low NKT cell numbers and decreased innate IFN-γ release have been associated with mycobacterial diseases (Yang et al., 2020) or with an enhanced susceptibility to infections with the Epstein Barr Virus (EBV) (Rigaud et al., 2006).

**Figure 2:**
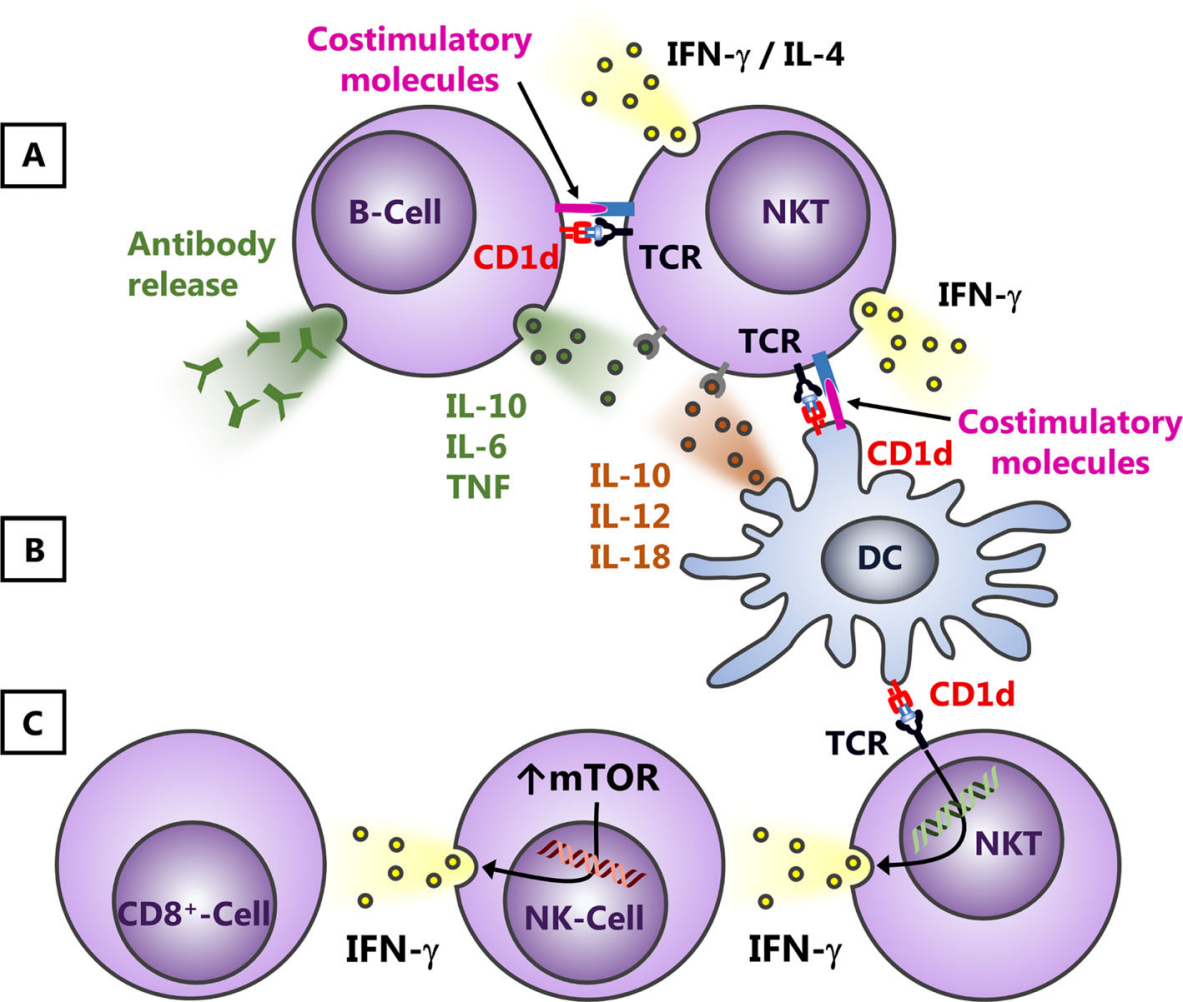
Effects of NKT cell activation on B-lymphocytes and NK cells. NKT cells and antigen presenting cells such as B-lymphocytes **(A)** or DCs **(B)** influence each other due to cognate and bystander activation. Thus, on the one hand, B-lymphocytes and DCs can modify NKT cell responses due to variations in the presentation of lipid antigens and/or an altered release of cytokines and/or changes in the expression of costimulatory molecules. B cells can promote thereby also Th2 responses **(A)**, whereas DCs presumably trigger predominantly the release of Th1 cytokines **(B)**. Vice versa, dependent on the interaction with cytokines, costimulatory molecules and/or the lipid antigens presented, NKT cells, for example, can alter their cytokine profile (Th1 and Th2) and subsequently suppress or augment B cell responses. This can affect the release and/or the class switch of antibodies by B-lymphocytes **(A)** or the cytokine profile and/or expression of costimulatory molecules by DCs **(B)**. Within a more Th1-dominated cytokine milieu, NKT cells can cross-activate NK cells and CD8^+^ T-lymphocytes **(C)**, a process that involves IFN-γ. This release of cytokines by NKT cells, however, does not only augment subsequent immune responses, but can also contribute to immunosuppression, for example, due to the upregulation of mTOR.

Following NKT cell activation, many other cell populations subsequently respond to the released cytokines, including different myeloid cell subsets, NK cells, T- and B-lymphocytes (Godfrey et al., 2004; Bendelac et al., 2007). This has been associated with immune cell activation and the augmentation of respective immune responses. Thus, lipid antigens activating NKT cells are utilized as adjuvants in various vaccination strategies. However, dependent on the expression of CD1d and the cytokine receptor expression profile (**Figure 2**), NKT cells can also suppress the downstream cellular network. For example, NKT cells interact with B cells or DCs through cognate or bystander interactions (Mattner et al., 2008; Chang et al., 2011; King et al., 2011) (**Figures 2A, B** **)**. Dominant influence of one or the other pathway might contribute to the observation that NKT cells can serve both as helpers for effector B-lymphocytes and negatively regulate autoreactive B cell responses (Sedimbi et al., 2020). Furthermore, an application of glyco-lipid containing nanoparticles enhances humoral immunity, but abrogates T cell - independent vaccine responses (Shute et al., 2021). IFN-γ released by NKT cells is also critical for the cross-activation of NK cells one the one hand (Carnaud et al., 1999) and the subsequent activation of CD8^+^ T cells. Indeed, NK cells are even the dominant cellular source for IFN-γ following NKT cell activation (Hoeksema et al., 2015; Kim et al., 2020). However, on the other hand, the release of cytokines by NKT cells interfering with NK cell responses can contribute also to immunosuppression due to the upregulation of mTOR (**Figure 2C**), as observed, for example, in bacterial sepsis (Kim et al., 2020).

## 5 Conclusion

In summary, several bacteria including *Sphingomonas*, *Borrelia*, *Streptococcus*, *Mycobacterium*, *Helicobacter*, *Corynebacterium* and *Bacteroides* express NKT cell antigens. These include GSLs and DAGs, which predominantly trigger the activation of NKT cells in a TCR-dependent manner when presented on CD1d. As these antigens are either highly immunogenic and/or present in large numbers in the bacterial cell wall, NKT cells can mediate a pivotal role for the clearance of these bacteria. Moreover, *Sphingomonas* spp., which are Gram-negative bacteria, express GSLs instead of LPS in their cell walls. Interestingly, one antigen of *Bacteroides fragilis* seems to inhibit the activation of NKT cells, which might contribute to bacterial immune evasion or reflect a mutual tolerance mechanism of NKT cells and commensal bacteria. As the TCR signal strength can also influence the polarization of NKT cell subsets (Tuttle et al., 2018), the functional phenotype of NKT cells, which tends to be imprinted in the thymus, might change even during the course of infection, particularly during chronic infections. Indeed, a functional polarization of NKT cells, similar to conventional T cells, can also occur during peripheral activation depending on the cytokine milieu characteristic of the specific activation context (Monteiro et al., 2010; Monteiro et al., 2013; Monteiro et al., 2015; Cameron and Godfrey, 2018). Akin to the more adaptive T cell response, NKT cells can expand and antigen-specific NKT cell clones can be generated that will assist in the clearance of infection. Thus, vice versa, microbial signals can also influence the functionality of NKT cells. Subsequently, further studies need to characterize the phenotypical changes of NKT cells in the respective infections and to delineate their functional consequences for bacterial clearance.

## Author Contributions

SV prepared the figure and added comments to the manuscript. JM wrote the manuscript. All authors contributed to the article and approved the submitted version.

## Funding

This study was supported by the Staedtler Stiftung (to JM), the German Research Foundation DFG (grant MA 2621/4-1 to JM, grant MA 2621/5-1 to JM and DFG-CRC 1181 - project number C04 to JM).

## Conflict of Interest

The authors declare that the research was conducted in the absence of any commercial or financial relationships that could be construed as a potential conflict of interest.

## Publisher’s Note

All claims expressed in this article are solely those of the authors and do not necessarily represent those of their affiliated organizations, or those of the publisher, the editors and the reviewers. Any product that may be evaluated in this article, or claim that may be made by its manufacturer, is not guaranteed or endorsed by the publisher.
